# Lecture upon Podophyllum

**Published:** 1888-06

**Authors:** J. T. Kent

**Affiliations:** Philadelphia


### LECTURE UPON PODOPHYLLUM.

PROFESSOR J. T. KENT, A. M., M. D., PHILADELPHIA.

This remedy is a proving of Jeanes. It is the mandrake. It has complaints peculiar to itself, like all other medicines. It affects the whole body, bringing out symptoms everywhere. Its symptoms are generally in connection with the portal system; we have diarrhœa and constipation in connection with this state of the portal system; when the diarrhœa is relieved, the head symptoms come on; when the constipation is relieved, the head symptoms come on; alternation of the diarrhœa or constipation with head and mind symptoms.

It has the natural gloominess that belongs to liver disorders; the liver is in a state of venous turgescence, hence we have in jaundice a melancholy. I should be disappointed if I did not find melancholy present in jaundice; that it should be absent would be peculiar. The alternation of the mental and abdominal symptoms is peculiar. The head symptoms will immediately subside, and on comes the diarrhœa; the diarrhœa will stop, and on comes a violent and indescribable headache, involving the greater part of the head. When you find that state don't forget Podophyllum. It has no equal in the *Materia Medica.*

In a general way abdominal plethora stands out strongly. The patient feels bloated, distended; there is soreness and uneasiness, all of which are relieved by stool.

Podophyllum produces a marked relaxation of all the pelvic structures and tissues, the rectum, vagina, and bladder; while urinating he feels as if the genital organs were pressing out; while urinating she feels as if the uterus would protrude; while at stool she feels as if the whole internal organs would protrude. Another marked evidence of this condition, prolapsus of the rectum while at stool from slightly straining, and from no straining at all. It seems to the patient as if the whole rectum protruded; this is associated with prolapsus uteri in the female, a horrible, indescribable feeling, as if the organs would fall out of the pelvic cavity. We have prolapsus of the uterus in other remedies, as Nux-v., *Sep.*, Murex, *Nat-m.* These are markedly prominent remedies for prolapsus uteri, but are no more prominent than Podoph. when indicated, and each has its own peculiar symptoms.

Suppose with this prolapsus you have a dragging feeling, a bearing down; with this there are pains, shooting pains, that shoot back to the rectum; every little twinge urges her to go to stool, or causes in her a desire to go to stool. You will give her Nux-v. and will cure. Nux-v. will also have frequent urging to stool and pains that shoot *from* the rectum. You will usually find other Nux-v. symptoms, such as are pretty apt to be in close relation to this phase of prolapsus by which you can decide. (This symptom also found under Plat., Aloe., Medor.)

How is it in Sepia? With this great dragging down and the uterus protruding, she *must* use a napkin to prevent the uterus from falling, or to prevent the sensation of falling. She is relieved by sitting or lying down; worse from walking; irresistible desire to press the uterus with the hand or napkin; aversion to man; to coition; with the associate symptoms of hot flushes; the constipation; the sense of lump in the rectum; the stool does not relieve; always a feeling as if fœcal matter remained in the rectum. With these associate symptoms you will surely give Sepia.

Murex has the dragging down like Sepia, even the protruding uterus. She has the strong desire to press the vulva with the hand or napkin, the *only* relief to her; she is *not* made better by lying down, like Sepia, but pains come on in the back and hips when lying down, compelling her to walk; walking increases the pain. Unlike Sepia, there is strong sexual desire; the sexual desire is increased; there are pains from the ovaries to the mammary glands; from the left ovary to the left mammary gland, and from the right ovary to the left mammary gland. All these remedies have greater or less engorgement of the pelvic region, tumidity, distended abdomen. The relaxation, want of tone of the veins, is most closely related to Podoph.

The complaints of Phodophyllum come on in the morning, commonly in the latter part of the night from two to four o'clock; we have a strong feature in the four o'clock chill and diarrhœa. What we have said in relation to chill and diarrhœa has been general.

Podoph. is of wonderful use in our summer complaints; complaints coming on because of the heat of the weather. The most characteristic of these diarrhœas is watery, yellowish, or dingy green, very profuse, mealy sediment. That is characteristic. The mother will say of her child, she does "not see where all the fluid comes from," it is so profuse; very offensive; pouring or gushing away. Phos.-ac. has this profuse gushing diarrhœa. How shall we tell them apart? In Podoph. we have deathly prostration; the child acts as if it were going to die; it has a pallid, deathly countenance. Phos.-ac has no prostration, and although the mother may say to you in the same way she does "not see where all the fluid comes from," she is surprised that baby is walking about. The Podoph. baby cannot do it.

In the congestion to the brain we have rolling of the head on the pillow associated with this diarrhœa. We have rolling of the head in Bell.; we have butting of the head, or lying with the head upon one side while the body is upon the back, in Apis. Bell. rolls the head in a congestion of the brain, where the brain is the primary seat of the disease, hence, we have rolling of the head in coma. In Podoph. it is in a congestion to the brain associated with bowel trouble. We commonly have profuse diarrhœa for a day or two, when it reaches a second stage; the diarrhœa is checked and on comes the head trouble, and the rolling of the head in the pillow. Here we have the alternation of the head and bowel symptoms.

Hunger after vomiting is prominent, and there is a great amount of vomiting. If you think Podophyllum does not vomit, get a half grain of the Podophyllum and take it; you will quite likely find out the facts through experience if you live to feel it. It produces profuse, greenish, watery vomiting; a vomiting of bile and frothy substance, and with the vomiting there is the deathly, overpowering prostration and nausea; he lies down a moment and then vomits again. Straining at stool or when vomiting produces prolapsus of the rectum and anus; he vomits everything; he vomits milk; this is not a characteristic, as when vomiting of milk is a characteristic it means milk and nothing else is vomited, and it becomes peculiar. The vomiting of milk is very common to Calc-c. and to Æthusa; the latter sometimes vomits milk and retains water. Æthusa is strongly characterized by the vomiting of milk. Prolapsus while vomiting, Mur-ac.

Podophyllum produces a habit of vomiting periodically; vomiting coming on in the morning at two or four o'clock, associated with gastric or duodenal catarrh, has found a useful remedy in Podoph. because of this periodicity. You will find patients who have it whenever they overload the stomach, or, if that is not the cause, they will have it every two weeks or so.

Fullness of the right hypochondrium, with associate symptoms of melancholy. Podoph. is a wonderful remedy for the mental symptoms associated with incurable organic disease of the liver; it improves the mental condition of the patient.

Now and then you will find a patient suffering from a diarrhœa that he has had for a long time; frequent, watery, yellowish stools, with the portal congestion belonging to, or similar to the Podoph. engorgement, hemorrhoids, etc. Podoph. will cure piles and all. The engorgement might remind you of Aloes, but you remember the cutting pains around the navel in Aloes; cutting pains while at stool, and a sensation of insecurity of the anus, also the jelly-like stool. Podoph. stool is more watery. Jelly-like stool is found under Colch., Coloc., Helle., Rhus, Sep., etc.

Another prominent symptom of Pod. is the undigested stool; the milk passes undigested; it is sometimes dry and chalky; the chalky stool is very prominent in Podoph. Calc-c. is prominent with chalky stool.

There is a great amount of flatus, with sputtering at stool, but not so marked as Aloes.

Another feature is a *painless* diarrhœa, but it is more common for the violent cutting colic to come and go, come and go; pains go off *with* stool and *without*. The urging is violent and sudden, and comes with a griping, boring pain; the inflammatory state following runs very high, producing great pain and engorgement.

With the painless diarrhœa we compare Chin. The diarrhœa of Chin. comes on at night and after eating; the Podoph. diarrhœa comes on in the morning and runs through the day; Chin. will stop during the day except after meals, and have a violent diarrhœa of an inky fluid at night. Podoph. is yellowish stool, very offensive, even putrid; Chin. is putrid, or sometimes not offensive and of inky color. It is rare for Podoph. to be indicated when the watery diarrhœa is not offensive, while China may be indicated.

Podophyllum is very useful in seasons of cholera, with profuse, watery, rice-water discharges, which, after *standing*, become jelly-like. The deathly sinking and prostration are such as are found in cholera.

Podophyllum has a great deal of chill, sweat, and some fever. Like its diarrhœa, it has a chill at four A. M. Coming in the morning, the diarrhœa may drive him out of bed, like Sulph. and Aloes. The sweat is most profuse about the head, especially the forehead. You will find this condition associated with the abdominal complaints and bowel troubles of Podoph.

The body is offensive; the perspiration is offensive.

Study and compare with Sep., Merc., Aloes, Sulph., and Nux-v. S. L. G. L.

